# Femoroacetabular impingement and its implications on range of motion: a case report

**DOI:** 10.1186/1752-1947-5-143

**Published:** 2011-04-10

**Authors:** Peter R Krekel, Anne JH Vochteloo, Rolf M Bloem, Rob GHH Nelissen

**Affiliations:** 1 Department of Orthopaedics, Leiden University Medical Center, Leiden, The Netherlands; 2 Computer Graphics, Delft University of Technology, Delft, The Netherlands; 3 Department of Orthopaedics, Reinier de Graaf Gasthuis, Delft, The Netherlands

## Abstract

**Introduction:**

Femoroacetabular impingement leads to limited hip motion, pain and progressive damage to the labrum. Assessment of the amount and location of excessive ossification can be difficult, and removal does not always lead to pain relief and an increase of function. One of the challenges ahead is to discover why certain cases have poor outcomes.

**Case presentation:**

The technical and clinical results of two consecutive arthroscopic shavings of an osseous cam protrusion are described in our patient, a 50-year-old Caucasian man with complaints of femoroacetabular impingement. At 12 weeks after the first arthroscopic shaving, our patient still experienced pain. Using a range of motion simulation system based on computed tomography images the kinematics of his hip joint were analyzed. Bone that limited range of motion was removed in a second arthroscopic procedure. At six months post-operatively our patient is almost pain free and has regained a range of motion to a functional level.

**Conclusion:**

This case demonstrates the relevance of range of motion simulation when the outcome of primary arthroscopic management is unsatisfactory. Such simulations may aid clinicians in determining the gain of a second operation. This claim is supported by the correlation of the simulations with clinical outcome, as shown in this case report.

## Introduction

In femoroacetabular impingement (FAI), deformations of the femoral head or the acetabular rim lead to bony impingement, resulting in limited hip motion, pain and progressive damage to the labrum. Although the etiology of FAI is still unclear, a variety of causes have been described, such as excessive sporting activities and post-traumatic or congenital deformities (for example, developmental dysplasia of the hip).

Two types of FAI are recognized: the cam type and the pincer type. When ossifications of the acetabular rim causes overcontainment of the hip, this is referred to as a pincer type FAI. Cam type FAI refers to deformations of the femoral head that reduce the head-neck offset. The two types have been reported to occur simultaneously in 86% of patients [1]. However, recent evidence indicates that cam and pincer hips are distinct pathoanatomic entities [2]. Treatment options vary, and include surgical dislocation and arthroscopic surgery. Satisfactory outcome is reported to range from 90% to 100% for arthroscopic management [3,4] and from 68% to 80% for open surgery [5,6].

Several measurements can be performed on plain radiographs and magnetic resonance imaging (MRI) to assess patients with FAI. The α angle that evaluates the prominence of the anterior femoral head-neck junction and the crossover sign for assessment of the amount of acetabular coverage are the most frequently used measurements [7]. Other measurements include the β angle, CE angle, anterior femoral distance and the femoral neck ratio [8-10]. To date, none of these measurements have been proven superior and as yet there is no gold standard to confirm FAI.

In the case of a failed primary surgical correction, the decision whether to perform secondary surgery is based on considerations regarding the altered expectations of the patient in combination with the limited chance of improvement. In addition, the risk of further weakening the femoroacetabular joint must be assessed, as it has been shown that bone strength is greatly affected when large amounts of bone are removed from the femoral head [11]. When the outcome of primary management is unsatisfactorily, this is frequently due to persisting impingement [4]. Additional evaluative instruments may support further treatment decisions.

In our case report, we describe the utilization of a range of motion (ROM) simulator to analyze the bone-determined ROM of a hip joint. From this analysis we learned which motion patterns might lead to FAI symptoms for our patient. This case demonstrates that analysis and simulation of computed tomography (CT) images can improve comprehension of the femoral head and its relation to the acetabular rim. The system can support surgeons in the decision whether or not to perform secondary surgery.

## Case presentation

Our patient was a 50-year-old Caucasian man with a history of a progressively worsening painful right hip for the last five years. His work and sports activities were limited due to the hip pain. His limping became more apparent over the last year, and the pain forced him to stop walking after five minutes.

A physical examination revealed pain with rotation at 90° of flexion. Flexion beyond 100° was not possible. Internal rotation was limited to 20°. External rotation was not impaired. An anterior impingement test was positive.

**Figure 1:**
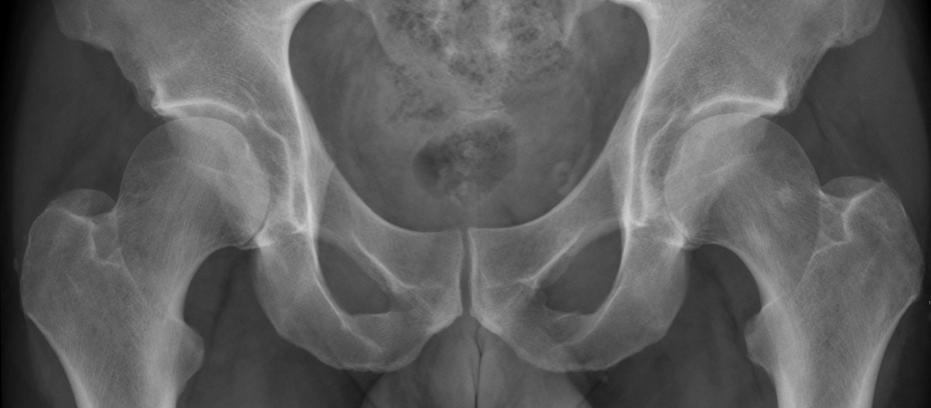
**Pre-operative anteroposterior and lateral radiographs**.

**Figure 2:**
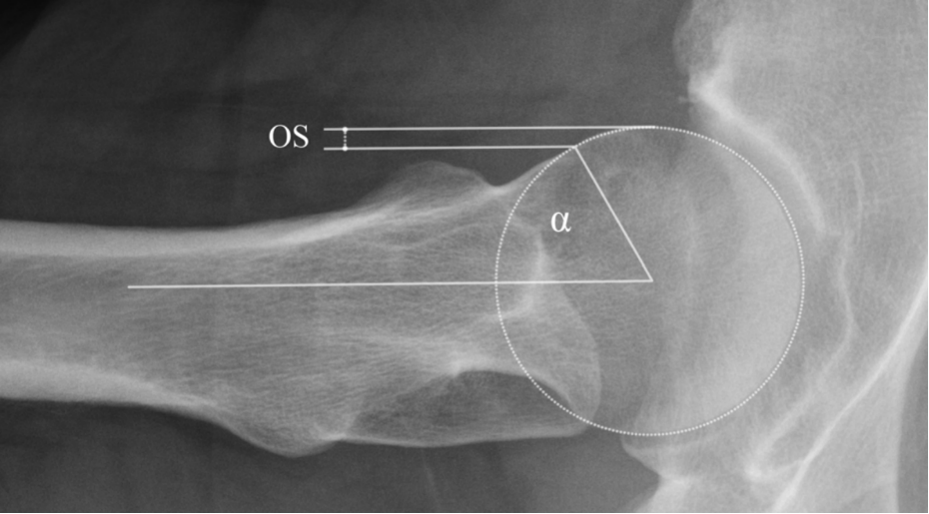
**Axial view of the femur, showing an increased α angle (62°) and decreased head-neck offset (OS)**.

Radiographs unveiled a cam deformity at the anterosuperior side of the femoral head and mild degenerative changes on the acetabular side (see Figures 1 and 2). The α angle was 60° (>55° is regarded abnormal and suspicious for a cam lesion [10]). The CE angle of 28° was within the normal range, and not larger than the cut-off point of 35° for a pincer type. An additional MRI scan did not reveal any labrum or cartilage pathology, or loose bodies. We agreed to perform an arthroscopy and shave the femoral head-neck junction if a cam lesion was found. During arthroscopy the suspected cam lesion was seen on the anterosuperior side of the femoral neck; additionally, we saw an intact labrum and mild degenerative changes of the cartilage of the anterolateral part of the acetabulum (Outerbridge classification I-II) [12]. The cam lesion was shaved off.

The arthroscopic correction was only marginally successful, as pain persisted 12 weeks after surgery. A minimal improvement in daily work and walking distance was seen. Using a regular CT scan we assessed whether a sufficient portion of the cam protrusion had been shaved off. This seemed to be the case on the regular, static CT images. Subsequently, it was decided to simulate the ROM of our patient in order to gain insight in the kinematics of the joint. Using Articulis (Clinical Graphics, Delft, The Netherlands), a system for the simulation of bone-determined ROM, the CT scan of the hip joint was analyzed. Articulis uses a collision detection algorithm and a kinematic model to describe the ROM of spherical joints such as the hip joint [13].

**Figure 3:**
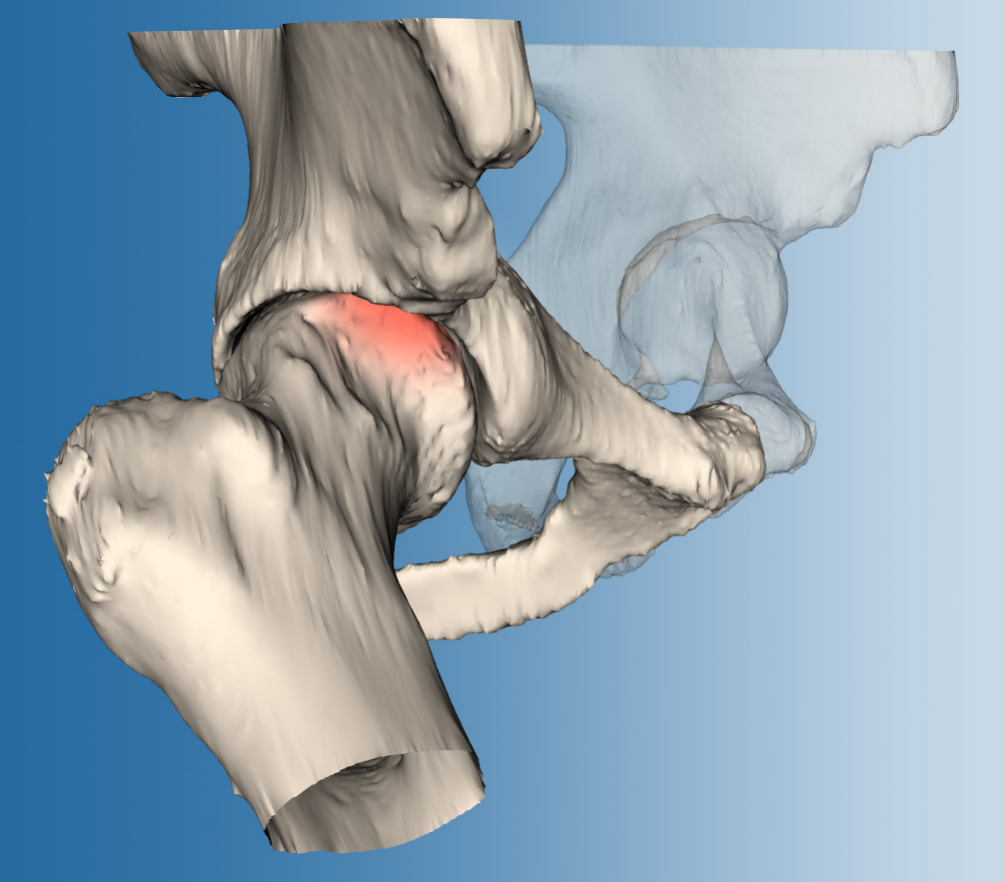
**Range of motion (ROM) simulations of the hip joint**. The pose of the femur is adjustable. When impingement is detected, the femur is colored red.

According to the simulations the risk of impingement was small in flexion and abduction separately. However, 45° of this combined motion was predicted to lead to impingement (see Figure 3). Internal rotation at 90° of flexion was limited to 15°, compared to 35° (±12°) in healthy hip joints as found by Tannast *et al*. [14].

**Figure 4:**
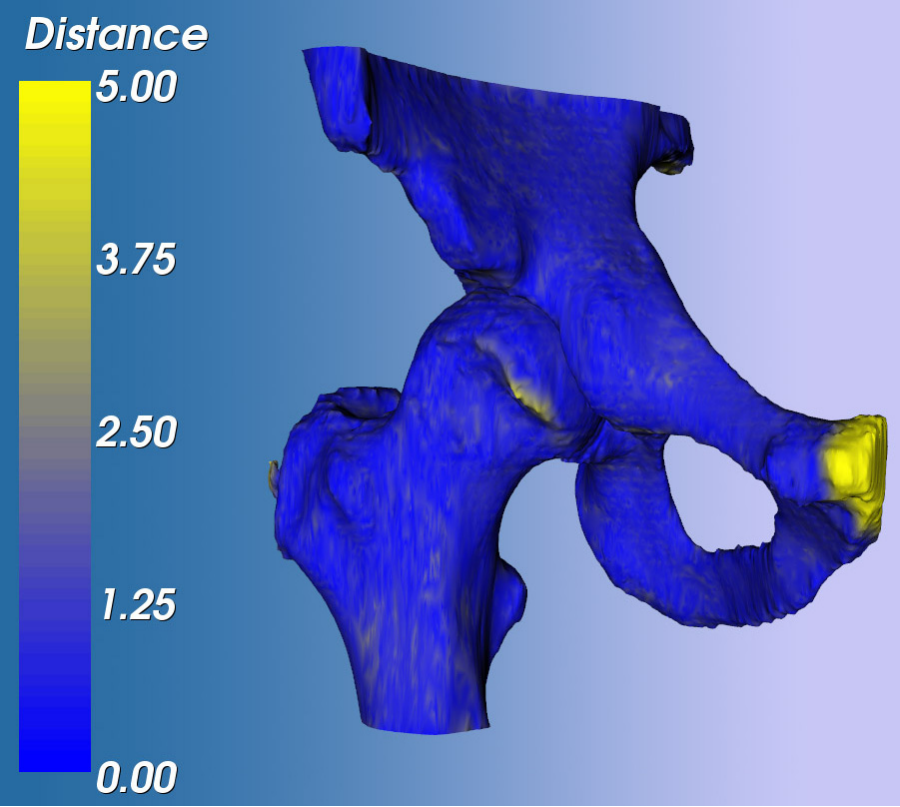
**Point-distance map of the femur and acetabulum after the first arthroscopic shaving**. The bone models are compared to the bone models extracted from the computed tomography (CT) scan that was performed after the second procedure. This visualization indicates which part of the femoral head has been shaved off during the second arthroscopy. Point distances are in mm. The pubis is colored yellow because of a difference in the scanned area.

**Figure 5:**
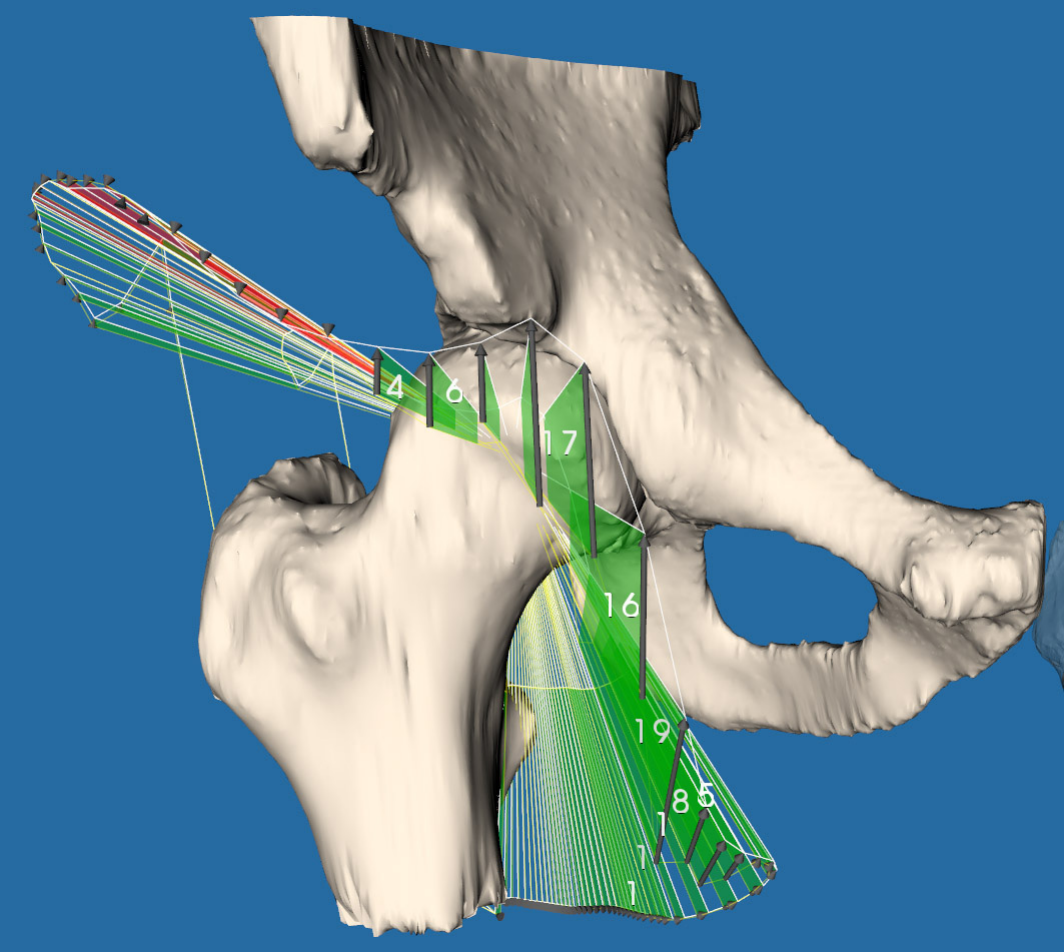
**Range of motion (ROM) simulation results using the post-operative computed tomography (CT) scan**. This visualization depicts the outlines of the ROM as constrained by collision between the two bones. The green surfaces depict the ROM improvement when compared to the bone models of the pre-operative CT scan. As can be seen, 19° of internal rotation was gained by further shaving of the femoral head.

We agreed upon a second arthroscopy of the affected hip. During this procedure, the remaining osseous rim on the femoral head was shaved off. At six months after this procedure, our patient is almost pain free and has regained a pain-free functional ROM. His limp has resolved and he can walk pain free. Informed consent for a CT scan was obtained to evaluate the last operation. This scan was analyzed using Articulis, and showed that bone-determined ROM had improved (see Figures 4 and 5).

## Discussion

Various methods to diagnose FAI have been described. Measurements on plain radiographs are generally performed on anteroposterior radiographs and an axial cross-table view of the proximal femur. These radiographic measurements are difficult to perform because of errors in projection, varying image contrast and misinterpretation of landmarks due to local osseous deformities. CT and MRI have been shown to represent an accurate alternative to quantify the femoral head-neck concavity. However, this only holds for the static case, whereas we believe that dynamical analysis of the joint is required to assess impingement.

Tannast *et al*. describe an extensively validated ROM simulation system that dynamically assesses the mobility of the femoroacetabular joint [15]. Our system is comparable to their system, although it was originally intended for impingement prediction in shoulder arthroplasty and was validated as such in a cadaveric study [13]. Still, the concept is similar, as a kinematic model approximating a spheroid joint is used in combination with collision detection algorithms to detect impingement. The objective of this study was to demonstrate that a dynamic ROM simulation system can show when and where impingement happens due to a remaining osseous rim after surgery. This can be helpful in deciding whether to perform a second operation.

Specific variations in the location of impingement have been described by Ito *et al*. [16]. The mean femoral head-neck offset was smaller in younger men on the anterior side (from lateral to medial), but in older women on the medial side (from anterior to anterolateral). The exact location of the deformity affects the spatial relation of the femoral head with respect to the acetabular rim. In addition, the high probability of a combination with a pincer type impingement complicates this spatial relation [1]. Appreciating these difficulties, analysis and simulation of ROM improves comprehension of the spatial relation of the femoral head and the acetabular rim. In more complicated cases with unsatisfactory primary results, three-dimensional motion analysis might be of even more help.

After primary surgery, 68% to 100% of the patients are satisfied with the result [3-6]. As indicated by Philippon *et al*. the reason of dissatisfaction in the majority of unsatisfied patients is probably caused by persisting impingement [4].

## Conclusion

Evaluation of the spatial relation between the femoral head and the acetabular rim in FAI requires precise imaging methods. In some cases, especially in cases where the surgical correction is insufficient, pathological deformities may be missed by conventional techniques and advanced techniques such as the three-dimensional simulation method described in this article may benefit the evaluation process. An important consideration in the decision for further treatment is that re-operation, whether arthroscopically or open, is difficult and burdensome, both for the patient and surgeon. Additional image modalities and simulation instruments that support and justify this decision are beneficial in this matter for both the surgeon and the patient.

In the case of our patient, the use of simulation software to establish how osseous anatomy disturbs function of the hip joint seems effective. The hypothesis is that it is a helpful tool in decision-making about treatment of FAI. Our model should be thoroughly tested in the future, using a randomized controlled trial to endorse the encouraging results described in our case report.

## Consent

Written informed consent was obtained from the patient for publication of this case report and any accompanying images. A copy of the written consent is available for review by the Editor-in-Chief of this journal.

## Competing interests

The authors declare that they have no competing interests.

## Authors' contributions

PRK developed the software application that was used to simulate range of motion. AJHV was a major contributor towards writing the manuscript. RMB was the surgeon who treated our patient, performed both arthroscopic operations and initiated the use of our assessment method. RGHHN was a major contributor towards writing the manuscript. All authors read and approved the final manuscript.
